# Supplementary material for: Free vibration characteristics of trapezoidal nanoplate rested on viscoelastic substrate with arbitrary boundary conditions using differential quadrature method
Source: PLoS One. 2025 Mar 31;20(3):e0316210. doi: 10.1371/journal.pone.0316210 (PMC11957281; doi:10.1371/journal.pone.0316210)
Supplement: Attachment — Submitted filename: Reviewer-Report-PONE-D-24-34233.docx [file pone.0316210.s001.docx]

**Reviewer Report**

**Manuscript Title:** Free Vibration Characteristics of Trapezoidal Nanoplate Rested on Viscoelastic Substrate With Arbitrary Boundary Conditions Using Differential Quadrature Method

The paper investigates the free vibration characteristics of trapezoidal nanoplates resting on a viscoelastic substrate using the first-order shear deformation theory (FSDT) and nonlocal elasticity theory, with the differential quadrature (DQ) method as the primary computational tool. This combination of methods addresses an important topic in the field of nanoelectromechanical systems (NEMS), especially in the design and analysis of nanosensors and resonators, as the results can provide significant insights into vibration characteristics at the nanoscale.

The problem is well-framed within the context of modern engineering challenges, such as the need for efficient, sensitive NEMS operating in damped environments. The following minor observations are suggested to be incorporated before considering the article for publication:

1.	The introduction could benefit from a more detailed explanation of the novelty and main contribution of the research, which is currently lacking. The authors should more clearly outline how their approach is different from previous works and why their focus on trapezoidal nanoplates specifically adds value to the existing body of knowledge.

2.	The literature review can be enhanced by incorporating recent studies and replacing outdated references where possible. Specifically, references 17, 18, 19, 35, and 37 can be updated with more current research unless they are essential to cite. To improve the relevance and impact of your references, I suggest replacing a few older studies with the following recent and relevant articles:

Zhang, J., & Zhang, C. (2023). Using viscoelastic materials to mitigate earthquake-induced pounding between adjacent frames with unequal height considering soil-structure interactions. Soil Dynamics and Earthquake Engineering, 172, 107988. doi: https://doi.org/10.1016/j.soildyn.2023.107988.

Du, G., Zhang, H., Yu, H., Hou, P., He, J., Cao, S.,... Ma, L. (2024). Study on Automatic Tracking System of Microwave Deicing Device for Railway Contact Wire. IEEE Transactions on Instrumentation and Measurement, 73, 1-11. doi: 10.1109/TIM.2024.3446638.

Cheng, Y., Fu, L., Hou, W., Carcione, J. M., Deng, W.,... Wang, Z. (2024). Thermo-poroelastic AVO modeling of Olkaria geothermal reservoirs. Geoenergy Science and Engineering, 241, 213166. doi: https://doi.org/10.1016/j.geoen.2024.213166.

3.	Despite the strengths, the discussion could benefit from a more thorough interpretation of the physical significance of these results. For instance, the role of boundary conditions in practical applications of NEMS is not fully explored. Additionally, the explanation of figures and tables is somewhat lacking in depth; more physical reasoning and detailed analysis would enhance clarity for readers.

4.	The conclusions adequately summarize the key findings, particularly the role of stiffness, nonlocal parameters, and damping in influencing natural frequencies. However, the conclusions are rather brief and could be strengthened by offering more in-depth insight into how the findings contribute to advancing the field of nanoscale vibration analysis.
