# Supplementary material for: Free vibration characteristics of trapezoidal nanoplate rested on viscoelastic substrate with arbitrary boundary conditions using differential quadrature method
Source: PLoS One. 2025 Mar 31;20(3):e0316210. doi: 10.1371/journal.pone.0316210 (PMC11957281; doi:10.1371/journal.pone.0316210)
Supplement: Attachment — Submitted filename: Reviewers Comments.docx [file pone.0316210.s002.docx]

**Title**

Free Vibration Characteristics of Trapezoidal Nanoplate Rested on Viscoelastic Substrate with Arbitrary Boundary Conditions Using Differential Quadrature Method

**Overview**

This study investigates the free vibration analysis of trapezoidal nanoplate resting on viscoelastic foundation based on first order shear deformation theory (FSDT) incorporating nonlocal elasticity theory, using differential quadrature (DQ) method.

**Comments**

- The paper is well-structured but requires further attention to grammar, orthography, and readability. There are some mistakes, sentences to be rewritten and long paragraphs that should be dealt with to improve clarity and comprehension.
- The main contribution of the work is not clearly stated. A clear statement summarizing the main contribution and its relevance to the field should be included in Section 1.
- A paragraph explaining the organization of the manuscript should be added at the end of introduction section. This will help guide readers through the structure of the paper.
- While looking at the broader spectrum of the research, the literature review provided in the manuscript is quite limited particularly in the context of vibration characteristics of structures considered therein. Therefore, I strongly suggest to update the literature review effectively thereby incorporating relevant recent studies to provide a stronger foundation for the research on free vibration characteristics of trapezoidal nanoplate resting on viscoelastic substrates. These recent works contribute into vibration behavior, modeling techniques, and applications, thereby enriching the manuscript.

To enhance understanding of vibration characteristics in complex systems, it is suggested to consider the investigation performed by Yuan et al. (2024), Chinese Journal of Mechanical Engineering, 37(1), 96 <https://doi.org/10.1186/s10033-024-01074-w>.

To leverage machine learning techniques for vibration analysis, it is vital to incorporate the reference of findings examined by Fan et al (2024) Automation in Construction, 167, 105694 10.1016/j.autcon.2024.105694.

To explore vibration isolation techniques using advanced structures, the work of Tian et al. (2024) Mechanical Systems and Signal Processing, 218, 111587 <https://doi.org/10.1016/j.ymssp.2024.111587>, on low-frequency vibration isolation using a concave X-shaped structure provides a useful reference for discussing vibration damping mechanisms, which could be particularly relevant when exploring the effects of the viscoelastic substrate in your analysis.

To address advanced force identification methods, Li et al. (2024) Structures, 60, 105840 [10.1016/j.istruc.2023.105840](http://dx.doi.org/10.1016/j.istruc.2023.105840), introduced a novel regularization method for identifying moving forces, which could be applied to refine the understanding of external dynamic forces in the nanoplate's vibration behavior.

To consider mechanical properties of structural components, the mechanical behavior of complex structures, as explored by Cao et al. (2024) Construction and Building Materials, 433, 136608 10.1016/j.conbuildmat.2024.136608, provides a useful comparison for understanding how material properties of the nanoplate and substrate interact to influence vibration responses.

To incorporate wave propagation and attenuation models, Wu et al. (2024) Engineering Structures, 315, 118480 <https://doi.org/10.1016/j.engstruct.2024.118480> developed an analytical solution for wave propagation in periodic structures, which could provide a theoretical basis for investigating how waves propagate and attenuate in trapezoidal nanoplates.

To incorporate advanced motion control techniques, the motion control strategies explored by Song et al. (2024) IEEE Transactions on Instrumentation and Measurement, 99, [10.1109/TIM.2024.3413202](http://dx.doi.org/10.1109/TIM.2024.3413202), could provide useful analogies for controlling and understanding vibration dynamics in mechanical structures, particularly in managing dynamic response in nanoplate systems."

To utilize data-driven optimization in structural design, Huang et al. (2022) Engineering Structures, 251, 113479 <https://doi.org/10.1016/j.engstruct.2021.113479> demonstrated the use of data-driven optimization for structural design, offering techniques that could be adapted to optimize the boundary conditions and material configurations of trapezoidal nanoplates for improved vibration characteristics.

- Is there any specific reason for not considering more particular boundary conditions like springs, and masses?
- On page 5, change “Mathematical Modeling” to “Mathematical modeling” for consistency. On page 7, ensure that “Mapping and Solution Procedure” is written as “Mapping and solution procedure” to maintain uniformity in the formatting of headings and subheadings. Additionally, correct typographical errors such as “Zhao et al.[17]” to “Zhao et al. [17]” on page 3, and provide only one space after “SLGS [26] to” on page 4.
