# Supplementary material for: Free vibration characteristics of trapezoidal nanoplate rested on viscoelastic substrate with arbitrary boundary conditions using differential quadrature method
Source: PLoS One. 2025 Mar 31;20(3):e0316210. doi: 10.1371/journal.pone.0316210 (PMC11957281; doi:10.1371/journal.pone.0316210)
Supplement: Attachment — Submitted filename: Response to reviewer PLOS ONE.docx [file pone.0316210.s003.docx]

**Manuscript number: PONE-D-24-34233**

**Title:** Free Vibration Characteristics of Trapezoidal Nanoplate Rested on Viscoelastic Substrate with Arbitrary Boundary Conditions Using Differential Quadrature Method

**Journal:** **PLOS ONE**

**Dear Editor-in-Chief, Dr. Emily Chenette**

Thank you for obtaining reviewers’ comments made on our manuscript. We have attempted to modify it accordingly, as highlighted in the revised manuscript. Detailed corrections are listed below point by point:

**# Reviewer 1**

*Reviewer’s comment:*

The paper investigates the free vibration characteristics of trapezoidal nanoplates resting on a viscoelastic substrate using the first-order shear deformation theory (FSDT) and nonlocal elasticity theory, with the differential quadrature (DQ) method as the primary computational tool. This combination of methods addresses an important topic in the field of nanoelectromechanical systems (NEMS), especially in the design and analysis of nanosensors and resonators, as the results can provide significant insights into vibration characteristics at the nanoscale.

The problem is well-framed within the context of modern engineering challenges, such as the need for efficient, sensitive NEMS operating in damped environments. The following **minor** observations are suggested to be incorporated before considering the article for publication:

1.	The introduction could benefit from a more detailed explanation of the novelty and main contribution of the research, which is currently lacking. The authors should more clearly outline how their approach is different from previous works and why their focus on trapezoidal nanoplates specifically adds value to the existing body of knowledge.

2.	The literature review can be enhanced by incorporating recent studies and replacing outdated references where possible. Specifically, references 17, 18, 19, 35, and 37 can be updated with more current research unless they are essential to cite. To improve the relevance and impact of your references, I suggest replacing a few older studies with the following recent and relevant articles:

- Zhang, J., & Zhang, C. (2023). Using viscoelastic materials to mitigate earthquake-induced pounding between adjacent frames with unequal height considering soil-structure interactions. Soil Dynamics and Earthquake Engineering, 172, 107988. doi: https://doi.org/10.1016/j.soildyn.2023.107988.
- Du, G., Zhang, H., Yu, H., Hou, P., He, J., Cao, S., Ma, L. (2024). Study on Automatic Tracking System of Microwave Deicing Device for Railway Contact Wire. IEEE Transactions on Instrumentation and Measurement, 73, 1-11. doi: 10.1109/TIM.2024.3446638.
- Cheng, Y., Fu, L., Hou, W., Carcione, J. M., Deng, W., Wang, Z. (2024). Thermo-poroelastic AVO modeling of Olkaria geothermal reservoirs. Geoenergy Science and Engineering, 241, 213166. doi: https://doi.org/10.1016/j.geoen.2024.213166.

3.	Despite the strengths, the discussion could benefit from a more thorough interpretation of the physical significance of these results. For instance, the role of boundary conditions in practical applications of NEMS is not fully explored. Additionally, the explanation of figures and tables is somewhat lacking in depth; more physical reasoning and detailed analysis would enhance clarity for readers.

4.	The conclusions adequately summarize the key findings, particularly the role of stiffness, nonlocal parameters, and damping in influencing natural frequencies. However, the conclusions are rather brief and could be strengthened by offering more in-depth insight into how the findings contribute to advancing the field of nanoscale vibration analysis.

*Author's answer:*

1. Thank you very much for your attention to the technical part of the study.

To the best of authors’ knowledge, no papers have been reported in the literature concerning the influence of viscoelastic substrate on vibrational behavior of the trapezoidal nanoplate resting on it. In the present study, the size dependent natural frequencies of trapezoidal nanoplate resting on viscoelastic foundation with different combinations of B.Cs have been conducted by means of FSDT and DQ method. The main contribution of the present study is considering the influences of viscoelastic medium in the examination of free vibration behaviour of trapezoidal-shaped nanoplates, having high shape diversity, with different combinations of Boundary conditions (B.Cs).

The reason of our focus on the new finding about trapezoidal shaped nanoplates, are:

Most of the researchers' studies have been investigated the vibration behavior of rectangular and circular nanosheets, and few works have been done on nanosheets with quadrilateral geometry. In 2017, Zhang et al. [1] succeeded in investigating the vibration behavior of nanosheets with a general shape under the effect of an external magnetic field using the Ritz free element numerical method. In this study, the effect of non-local parameter, geometrical angle and boundary conditions on free vibration behavior of parallelogram graphene sheet was investigated. In addition, 12 different states of a single-layer graphene sheet with general geometry were modeled and presented in different boundary conditions (as shown in the next figure).

|  |  |
| --- | --- |

Figure R1: Schematic of different geometries investigated from the quadrilateral graphene sheet [1]

2.Thank you so much for your kind attention to our study. As mentioned, we remove the older studies (references 17, 18, 19, 35, and 37) and replaced them with never papers, as follows:

- Nguyen Thi et al. [2] (2024) https://doi.org/[10.1016/j.heliyon.2024.e26150](http://doi.org/10.1016/j.heliyon.2024.e26150)
- Thom et al.[3] (2024) <https://doi.org/10.1016/j.euromechsol.2024.105309>
- Torabi et al. [4] (2019) <https://doi.org/10.1016/j.euromechsol.2018.07.009>
- Wang and Liu [5] (2024) <https://doi.org/10.1371/journal.pone.0308245>

Moreover, as you noticed the manuscript should be update by replacing new papers in the fields of system’s vibration. The three suggested papers, were in the fields of vibration and published in 2024, added to the manuscript as Refs [37, 38, 46] and highlighted in the revised version.

3. Thank you for your attention to our study. In the case of “interpretation of the physical consequence of the numerical results, such as the role of boundary conditions in practical applications of NEMS”, we add following sentences:

For example: at two different boundary conditions, CFCF (bridge-type) and CFFF (cantilever-type); Based on Table 4, the fundamental natural frequencies of CFCF have been reduced about 84% by removing one clamped BC. Consequently, type of BCs is very crucial in the free vibration behaviour.

Also, we add more physical reasoning and detailed analysis of Table and Figures, as follows:

- Figure 6: Based on the numerical results by increasing foundation’s springs parameter (K) the rigidity of the structure, and the natural frequency rises. Actually, resistance against deformation for the nanoplate resting on the foundation became stronger due to increasing of the elastic foundation stiffness. In other word, the nanoplate needs to overcome larger resistance to oscillate, so vibrates at a higher frequency.
- Figure 5: By increasing the foundation damping, the frequency curves reduced nonlinearly, which eventually led to zero frequencies and overdamped state. At this condition if the damping parameter increased and became more than a critical damping parameter then the nanoplates did not oscillate. The critical damping parameters are depended on considered nonlocal parameters; for example, at μ=0.1, critical damping equals to C_cr_ = 22.
- Fig. 4: Based on the Fig.4, by increasing the nonlocal parameter and soften the nanoplates, the natural frequencies of nanoplate decreased.
- Fig. 3: It can be decided that as the nanoplate has been transformed from oblique (skewed)shape to the rectangular shape (with β=90), the natural frequency decreases, considerably.
- Table 4: Based on the results, the fundamental natural frequencies of CFCF have been reduced about 84% by removing one clamped BC. Consequently, type of BCs is very crucial in the free vibration behaviour.
- Table 3: The frequency value drops significantly in the plates with replacing clamped boundary with free boundary.
- Table 2: By increasing the h/a ratio, the frequency parameter of all modes decreased.

4. Based on your appropriate comment, about “How the findings contribute to advancing the field of nanoscale vibration analysis? “, we complete the conclusion part, as follows:

The results indicated that adjusting the foundation parameters and BC type of nanoplate can be effective tactics in modifying the vibration behavior of the system. Changing these factors could critically affect vibration features, signifying the opportunity of operating these nano- structures for numerous NEMS applications with specific functionality, such as vibration damping. Also, the new findings can be utilized as a benchmark for upcoming studies about use of quadrilateral nanoplates in real-world engineering scenarios (like nano-sensors, nano-resonators, nano-switches and nanoscale devices working at high frequencies); for example, more particular boundary conditions like elastic BCs with transverse and rotational springs.

**# Reviewer 2**

*Reviewer’s comment:*

**Overview**

This study investigates the free vibration analysis of trapezoidal nanoplate resting on viscoelastic foundation based on first order shear deformation theory (FSDT) incorporating nonlocal elasticity theory, using differential quadrature (DQ) method.

**Comments**

1. The paper is well-structured but requires further attention to grammar, orthography, and readability. There are some mistakes, sentences to be rewritten and long paragraphs that should be dealt with to improve clarity and comprehension.
2. The main contribution of the work is not clearly stated. A clear statement summarizing the main contribution and its relevance to the field should be included in Section 1.
3. A paragraph explaining the organization of the manuscript should be added at the end of introduction section. This will help guide readers through the structure of the paper.
4. While looking at the broader spectrum of the research, the literature review provided in the manuscript is quite limited particularly in the context of vibration characteristics of structures considered therein. Therefore, I strongly suggest to update the literature review effectively thereby incorporating relevant recent studies to provide a stronger foundation for the research on free vibration characteristics of trapezoidal nanoplate resting on viscoelastic substrates. These recent works contribute into vibration behavior, modeling techniques, and applications, thereby enriching the manuscript.

- To enhance understanding of vibration characteristics in complex systems, it is suggested to consider the investigation performed by Yuan et al. (2024), Chinese Journal of Mechanical Engineering, 37(1), 96 <https://doi.org/10.1186/s10033-024-01074-w>.
- To leverage machine learning techniques for vibration analysis, it is vital to incorporate the reference of findings examined by Fan et al (2024) Automation in Construction, 167, 105694 https://doi.org/10.1016/j.autcon.2024.105694.
- To explore vibration isolation techniques using advanced structures, the work of Tian et al. (2024) Mechanical Systems and Signal Processing, 218, 111587 <https://doi.org/10.1016/j.ymssp.2024.111587>, on low-frequency vibration isolation using a concave X-shaped structure provides a useful reference for discussing vibration damping mechanisms, which could be particularly relevant when exploring the effects of the viscoelastic substrate in your analysis.
- To address advanced force identification methods, Li et al. (2024) Structures, 60, 105840 https://doi.org/[10.1016/j.istruc.2023.105840](http://dx.doi.org/10.1016/j.istruc.2023.105840), offered a novel regularization method for identifying moving forces, which could be applied to refine the understanding of external dynamic forces in the nanoplate's vibration behavior.
- To consider mechanical properties of structural components, the mechanical behavior of complex structures, as explored by Cao et al. (2024) Construction and Build. Materials, 433, 136608 https://doi.org/10.1016/j.conbuildmat.2024.136608, provides a useful comparison for understanding how material properties of the nanoplate and substrate interact to influence vibration responses.
- To incorporate wave propagation and attenuation models, Wu et al. (2024) Eng. Structures, 315, 118480 <https://doi.org/10.1016/j.engstruct.2024.118480> developed an analytical solution for wave propagation in periodic structures, which could provide a theoretical basis for investigating how waves propagate and attenuate in trapezoidal nanoplates.
- To incorporate advanced motion control techniques, the motion control strategies explored by Song et al. (2024) IEEE Transactions on Instrumentation and Measurement, 99, https://doi.org/[10.1109/TIM.2024.3413202](http://dx.doi.org/10.1109/TIM.2024.3413202), could provide useful analogies for controlling and understanding vibration dynamics in mechanical structures, particularly in managing dynamic response in nanoplate systems."
- To utilize data-driven optimization in structural design, Huang et al. (2022) Eng. Structures, 251, 113479 <https://doi.org/10.1016/j.engstruct.2021.113479> established the use of data-driven optimization for structural design, offering techniques that could be adapted to optimize the boundary conditions and material configurations of trapezoidal nanoplates for improved vibration characteristics.

5. Is there any specific reason for not considering more particular boundary conditions like springs, and masses?
6. On page 5, change “Mathematical Modeling” to “Mathematical modeling” for consistency. On page 7, ensure that “Mapping and Solution Procedure” is written as “Mapping and solution procedure” to maintain uniformity in the formatting of headings and subheadings. Also, correct typographical errors such as “Zhao et al.[17]” to “Zhao et al. [17]” on page 3, and provide only one space after “SLGS [26] to” on page 4.

*Author's answer:*

1. All of the writing mistakes in the sentences, have been corrected and replaced in the revised manuscript file.

2.The main contribution of the present study is considering the influences of viscoelastic foundation in the examination of free vibration behaviour of trapezoidal-shaped nanoplates, having high shape diversity, with different combinations of Boundary conditions.

3. Thank you for your attention to our study. As you noticed, we add a paragraph explaining the organization of the study at the end of introduction section, as follows:

The present study is organized into four primary sections. Section 2 illustrated the mathematical modeling of nanoplate, the constructive relations and its governing equations of motion. These governing equations have been transformed from the trapezoidal physical domain into the rectangular computational domain in the Section 3 by means of mathematical operations and then rewritten in the form of generalized eigenvalue problem by means of DQ method for various B.C. Finally at section 4, the validation of numerical results presented and parametric study have been shown by means of plots.

4. While thanking your Excellency, as you noticed the manuscript should be update by replacing new papers in the fields of system’s vibration. The eight suggested papers, were in the fields of vibration and published in 2024, added to the manuscript as Refs [39-45, 47] and highlighted in the revised version.

Also, we decided to add another new study in the fields of nanostructure’s vibration for various application as follows:

- Nguyen Thi et al. [2] (2024) https://doi.org/[10.1016/j.heliyon.2024.e26150](http://doi.org/10.1016/j.heliyon.2024.e26150)
- Thom et al.[3] (2024) <https://doi.org/10.1016/j.euromechsol.2024.105309>
- Torabi et al. [4] (2019) <https://doi.org/10.1016/j.euromechsol.2018.07.009>
- Wang and Liu [5] (2024) <https://doi.org/10.1371/journal.pone.0308245>

5. Based on your appropriate comment; for the next study we planned to considered general elastic boundary condition for nanoplate with stiffness elements (rotational and transverse springs) in the all directions.

6. Thank you for your attention to our study; all of the mentioned errors have been corrected and highlighted in the revised manuscript file.

***References***

[1]	L. W. Zhang, Y. Zhang, and K. M. Liew, “Vibration analysis of quadrilateral graphene sheets subjected to an in-plane magnetic field based on nonlocal elasticity theory,” *Compos. Part B Eng.*, vol. 118, pp. 96–103, 2017.

[2]	T.-H. N. Thi, V. K. Tran, and Q. H. Pham, “Flexoelectric and size-dependent effects on hygro-thermal vibration of variable thickness fluid-infiltrated porous metal foam nanoplates,” *Heliyon*, vol. 10, no. 4, 2024.

[3]	D. Van Thom, V. M. Chinh, P. Van Minh, and N. D. A. Vu, “Mechanical responses of nanoplates resting on viscoelastic foundations in multi-physical environments,” *Eur. J. Mech. - A/Solids*, vol. 106, no. 105309, 2024.

[4]	J. Torabi, R. Ansari, and R. Hassani, “Numerical study on the thermal buckling analysis of CNT-reinforced composite plates with different shapes based on the higher-order shear deformation theory,” *Eur. J. Mech.*, vol. 73, pp. 144–160, 2019.

[5]	C. Wang and Q. Liu, “Buckling behaviour of rectangular and skew plates with elastically restrained edges under non-uniform mechanical edge loading,” *PLoS One*, vol. 19, no. 9, p. e0308245, 2024.

The revised manuscript has been submitted to your journal.

Best regards,

**Paweł Pławiak**

Corresponding author
